# The Colonisation of Calves in Czech Large-Scale Dairy Farms by Clonally-Related *Clostridioides difficile* of the Sequence Type 11 Represented by Ribotypes 033 and 126

**DOI:** 10.3390/microorganisms8060901

**Published:** 2020-06-15

**Authors:** Martina Masarikova, Ivana Simkova, Martin Plesko, Veronika Eretova, Marcela Krutova, Alois Cizek

**Affiliations:** 1 Department of Infectious Diseases and Microbiology, Faculty of Veterinary Medicine, University of Veterinary and Pharmaceutical Sciences, 612 42 Brno, Czech Republic; masarikovam@vfu.cz (M.M.); cizeka@vfu.cz (A.C.); 2 Ruminant and Swine Clinic, Faculty of Veterinary Medicine, University of Veterinary and Pharmaceutical Sciences, 612 42 Brno, Czech Republic; simkovai@vfu.cz (I.S.); pleskom@vfu.cz (M.P.); 3 Department of Medical Microbiology, 2nd Faculty of Medicine and Motol University Hospital, Charles University, 150 06 Prague, Czech Republic; eretovave@natur.cuni.cz

**Keywords:** calves, *Clostridioides difficile*, digestate, ribotype 033, ribotype 126, Thr82Ile, Holstein

## Abstract

To investigate a possible *Clostridioides difficile* reservoir in the Czech Republic, we performed a study in 297 calves from 29 large-scale dairy farms. After enrichment, faecal samples were inoculated onto selective agar for *C. difficile*. From the 297 samples, 44 *C. difficile* isolates were cultured (prevalence of 14.8%, 10 farms). The Holstein breed and use of digestate were associated with *C. difficile* colonisation (*p* ˂ 0.05). *C. difficile* isolates belonged to the ribotype/sequence type: RT033/ST11 (*n* = 37), RT126/ST11 (*n* = 6) and RT046/ST35 (*n* = 1). A multiple-locus variable-number tandem-repeat analysis revealed four clonal complexes of RT033 isolates and one clonal complex of RT126 isolates. All isolates were sensitive to amoxicillin, metronidazole and vancomycin. Forty isolates were resistant to ciprofloxacin, twenty-one to clindamycin, seven to erythromycin, seven to tetracycline and six to moxifloxacin. Moxifloxacin resistant isolates revealed an amino-acid substitution Thr82Ile in the GyrA. In conclusion, the calves of Holstein breed from farms using digestate as a product of bio-gas plants are more likely to be colonised by clonally-related *C. difficile* of ST 11 represented by ribotypes 033 and 126. The identified resistance to moxifloxacin with a Thr82Ile substitution in the GyrA highlights the need for further monitoring by the “One health approach”.

## 1. Introduction

*Clostridium difficile*, recently reclassified as *Clostridioides difficile*, is an anaerobic, a gram-positive, spore-forming bacterium [1]. *C. difficile* is a leading pathogen of healthcare-associated diarrhoea in humans and responsible for more than 152,000 reported *C. difficile* infections (CDI) and more than 8300 associated deaths every year in the European Union and European Economic Area (EU/EEA) [2].

Recently, it has been recognized that the prevalence of CDI is underestimated in the community [3]. The increasing significance of community-associated CDI has continuously stimulated efforts to find and clarify possible sources/reservoirs for human infection [4]. Research focused on the presence of *C. difficile* in the human food chain indicates that livestock could be a potential primary reservoir for the contamination of food products, the abundance of *C. difficile* in the environment and subsequent human gut colonization [5,6,7].

In livestock, calves are frequently colonized by *C. difficile* [8,9,10,11,12,13,14]. Surprisingly, the spectrum of identified genotypes is very similar within countries as well as continents. The most frequent ribotypes (RTs) identified in calf faeces were 078, 126 and 033 in Europe and Asia [9,11,13] and 127 and 288 in Australia [8]. These ribotypes belong to the same sequence type and clade (11/5) but differ in toxin gene profiles [8,15]. RTs 078, 126 and 127 carry genes for all three toxins (A, B and binary) but RTs 033 and 288 encode genes for the binary toxin and in some RT 033 strains, a part of the *tcdA* gene was detected [8,16].

The *C. difficile* ribotypes identified in calves overlap with *C. difficile* ribotypes recovered from human CDI samples, moreover, ribotype 078, the agriculture-related ribotype, belonged to the ten most frequent ribotypes in European hospitalised patients with CDI; except for the Eastern part of the continent [17,18].

To gather data on the prevalence of *C. difficile* in calves in Eastern Europe, a large-scale study of dairy farms was performed across the Czech Republic with a detailed characterisation of the *C. difficile* isolates obtained.

## 2. Materials and Methods

### 2.1. Characterization of Farms

Between June and September 2019, twenty-nine dairy farms across the Czech Republic participated in the study. At each farm, information on herd size, breed, housing type, milk production, calf rearing, type of bedding and the presence of a bio-gas plant were collected. The characteristics of the farms are summarized in Supplementary Table S1.

### 2.2. Clostridioides difficile Culture

Enrichment culture was performed in all faecal samples as described previously [19]. Approximately 0.5 g of faeces, were inoculated into cyloserine-cefoxitin fructose enrichment broth (Oxoid, Basingstoke, UK) supplemented with 0.1% sodium taurocholate (Sigma-Aldrich, St. Louis, MO, USA) and incubated at 37 °C for 7 days in an anaerobic chamber Concept 300 (Ruskinn Technology, Bridgend, UK). Thereafter, 1 mL of enrichment broth was mixed with 1mL of absolute ethanol and left for 1 h at room temperature. Finally, tubes were centrifuged at 1520× *g* for 10 min and the sediment plated onto solid selective medium selective agar chromID™ *C. difficile* (bioMérieux, Lyon, France). Inoculated plates were cultured under anaerobic conditions for 48 h at 37 °C. Suspected *C. difficile* colonies were identified using matrix assisted laser desorption/ionization time-of-flight mass spectrometry (MALDI-TOF MS) using a MALDI Biotyper v 3.0 system (Bruker Daltonics, Bremen, Germany).

### 2.3. Antimicrobial Susceptibility Testing and the Detection of Antimicrobial Resistance Determinants

The minimum inhibitory concentration (MIC) for amoxicillin, ciprofloxacin, clindamycin, erythromycin, metronidazole, moxifloxacin, tetracycline and vancomycin were determined by using the E-test (bioMérieux, Lyon, France) on Brucella blood agar (Oxoid, Basingstoke, UK) containing hemin (5 mg/mL) and vitamin K1 (10 mg/mL). The MIC breakpoints for metronidazole (2 mg/L), vancomycin (2 mg/L) and moxifloxacin (4 mg/L) were applied as recommended by the European Committee on Antimicrobial Susceptibility Testing (EUCAST), [20]. The MIC breakpoints for amoxicillin (16 mg/L), ciprofloxacin (4 mg/L), clindamycin (8 mg/L), tetracycline (8 mg/L) and erythromycin (8 mg/L) were determined according to the Clinical and Laboratory Standards Institute guidelines (CLSI) breakpoints for the susceptibility testing of anaerobic bacteria [21].

The presence of known antimicrobial-resistance determinants for tetracycline (*tetM*, *tetW*, *tetO*, *tetA/B*, *tet 0/32/0* and *tet44*), clindamycin/macrolides (*ermB*) and fluoroquinolones (*gyrA* mutations in the quinolone resistance-determining region (QRDR)) was investigated by PCR amplification and Sanger sequencing as previously described [17,19,22,23,24]. The primers used in the study are listed in Supplementary Table S2. 

### 2.4. Capillary Electrophoresis PCR Ribotyping, Multi-Locus Sequence Typing (MLST), Multiple-Locus Variable-Number Tandem-Repeat Analysis (MLVA) and Toxin Genes Detection

To distinguish between individual *C. difficile* isolates, the capillary electrophoresis (CE) PCR ribotyping was performed according to the consensus PCR ribotyping protocol [25]. The CE-ribotyping profiles were compared in a WEBRIBO database [26].

Further, the sequence type (ST) and clade were determined by comparing the sequences of seven housekeeping genes (*adk*, *atpA*, *dxr*, *glyA*, *recA*, *sodA* and *tpi*) in a MLST database (available at: http://pubmlst.org/cdifficile), [27,28].

The detection of toxin genes (*tcdA*-toxin A), (*tcdB*-toxin B) and (*cdtA*/*cdtB*-binary toxin) was performed by multiplex PCR reaction [29]. Of note, the *tcdA* truncated *C. difficile* strains could not be identified because the location of the primers for *tcdA* detection is upstream of the repetitive region.

To determine the genetic relatedness of *C. difficile* isolates belonging to the same ribotype and/or sequence type a MLVA was performed. Due to the absence of the A6Cd locus, six loci with short tandem repeats were used for the MLVA (B7Cd, C6Cd, E7Cd, F3Cd, G8Cd and H9Cd), [30]. A minimum spanning tree (MST) was created by Bionumerics v. 5.0 (Applied Math, bioMérieux, Lyon, France) using the Manhattan coefficient. A clonal complex (CC) was defined as the sum of tandem repeat differences (STRD) ≤ 2 [30].

### 2.5. Statistical Analysis

Data on breeding conditions were analysed using Fisher’s Exact Probability test. A *p*-value of ≤0.05 was considered statistically significant. A univariate logistic regression model was used for the analyses of associations between individual variables and outcomes of interest. Analyses were conducted using the UNISTAT v. 6.5.

## 3. Results

### 3.1. The Breed and Use of Digestate Is Associated with C. difficile Colonisation in Calves

**Figure 1:**
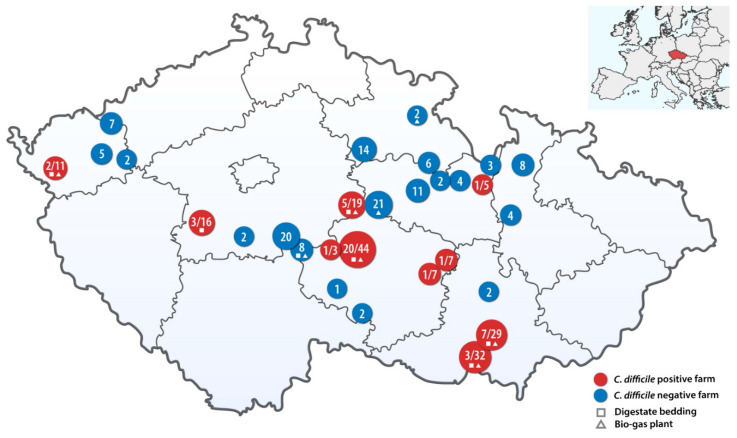
Geographic distribution of farms in the study. Red circles represent farms with the occurrence of *C. difficile* (number of positive/number of samples tested). Blue circles represent farms without the occurrence of *C. difficile* with number of samples tested.

A total of 29 dairy farms across the Czech Republic participated in the study (their geographic location is shown in Figure 1). The mean size of the dairy farm was 348 cows (range 15–1280, median 270 cows). The mean age of the investigated calves was 26 days (range 1–93 days, median 23 days). The breed distribution was as follows: Czech Fleckvieh 19 farms, Holstein 7 farms, crossbred in two farms and Montbéliarde in one farm. A bio-gas plant was operated in 8 farms and the digestate was used as bedding in 7 farms. 

A total of 297 calves were investigated, 20 of them (6.7%) had diarrhoea. *C. difficile* was cultured after enrichment from 44 samples; the overall prevalence of *C. difficile* was 14.8% in 10 farms (Figure 1). The mean age of the *C. difficile* positive calves was 18 days (range 2–58 days, median 14 days). No diarrhoeic calf was *C. difficile* positive.

The prevalence of *C. difficile* carriers among the calves ranged from 9.4 to 45.5% between farms (Table 1). Five of the ten *C. difficile*-positive dairy farms operate a bio-gas plant (*p* = 0.08) and six *C. difficile*-positive farms use digestate from this technology as bedding for cows (OR 27, 95% CI 2.5–291.2; *p* = 0.0026). The Holstein breed was more likely to be colonised by *C. difficile* (OR 8.5, 95% CI 1.25–57.93; *p* = 0.0302) (Table 2). Risk factors associated with *C. difficile* colonisation are summarized in Table 2.

### 3.2. Calves in Large Czech Dairy Farms Are Colonized by Clonally-Related Clostridioides difficile of the Sequence Type 11 Represented by the Ribotypes 033 and 126

Forty-four *C. difficile* isolates belonged to three different ribotypes (RT) and to two sequence types (ST), RT033/ST11 (*n* = 37), RT126/ST11 (*n* = 6) and RT046/ST35 (*n* = 1). *C. difficile* isolates of RT033 showed a positive PCR result for toxin A gene (*tcdA*) and binary toxin genes (*cdtA*/*B*); RT126 isolates carried all four toxin genes (*tcdA*, *tcdB*, *ctdA*/*B*) and the *C. difficile* isolate of RT046 carried genes for toxins A/B. A summary of results of *C. difficile* characterisation is shown in Table 1.

**Figure 2:**
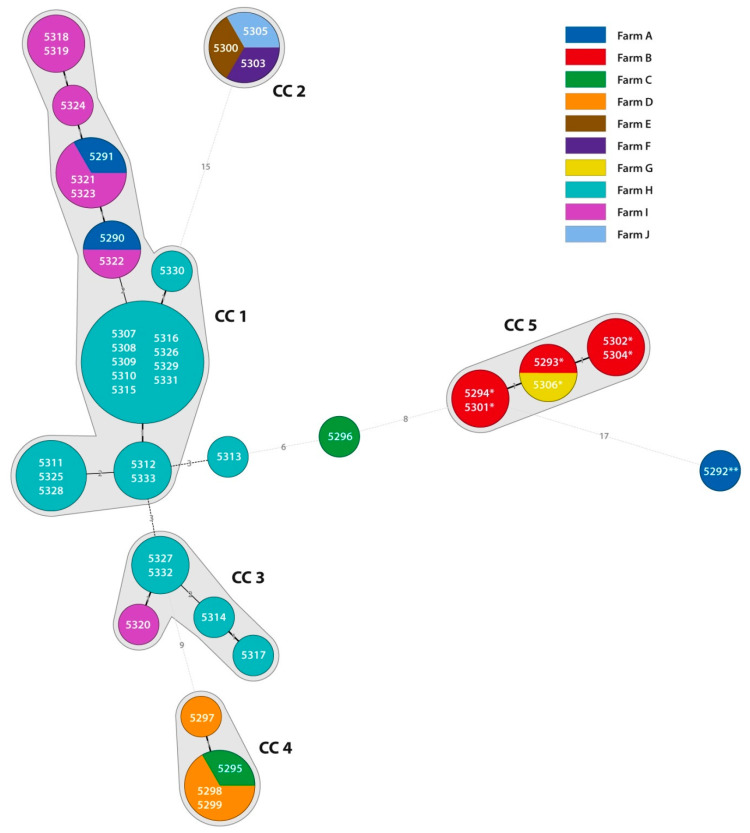
Minimum spanning tree of *Clostridioides* (*Clostridium*) *difficile* isolates in the study. The numbers in the circles represent DNA number of *C. difficile* isolate. The numbers on the lines represent the sum of tandem repeat differences (STRD) between isolates. If more than one number is present in one circle, it represents isolates with STRD = 0. A clonal complex (CC) was defined as the sum of tandem repeat differences (STRD) ≤ 2. * The clonal complex 5 consists of *C. difficile* isolates ribotype 126; ** The *C. difficile* isolate no. 5292 belongs to the ribotype 046.

A MLVA revealed four clonal complexes (CC) of RT033 isolates. The first CC consisted of 23 isolates (three farms), the second of three isolates (three farms), the third of 5 isolates (two farms) and the fourth of 4 isolates (two farms). The remaining clonal complex consisted of six RT126 isolates derived from two farms (Figure 2).

### 3.3. Ciprofloxacin and Clindamycin Resistance Predominated and Moxifloxacin Resistance Due to Thr82Ile Emerged

All isolates were sensitive to amoxicillin, metronidazole and vancomycin. Ten *C. difficile* isolates (22.7%) were multidrug resistant. Forty isolates (90.9%) were resistant to ciprofloxacin, twenty-one (47.7%) to clindamycin, seven to erythromycin (15.9%), seven to tetracycline (15.9%) and six to moxifloxacin (Table 2, Supplementary Table S3).

The isolate of RT 046 exhibited a high level of resistance to erythromycin and clindamycin (256 mg/L) and carried the *ermB*, an adenine methylase gene. All six moxifloxacin-resistant isolates revealed a Thr82Ile amino-acid substitution in the GyrA; moxifloxain-susceptible isolates were wild types in the sequences of the *gyrA* QRDR region. Of the seven *C. difficile* isolates resistant to tetracycline, three isolates carried *tetA* and *tetB*, the genes for tetracycline efflux transporters. 

## 4. Discussion

In cattle, *C. difficile* has greater significance as a zoonotic disease than as an animal pathogen [31]. The intestinal tract of cattle can serve as a primary source for environmental contamination by the spores of *C. difficile*, which survive very well in unfavourable conditions. Through manure and farm waste, the spores may be transmitted and spread to outside farms [32].

The prevalence of *C. difficile* in calves ranged between 1.8 to 22.2% [32], the prevalence detected may be influenced by the breeding conditions, the age of calves investigated and also by the method used for the detection of *C. difficile*. In our study, the *C. difficile* prevalence based on selective enrichment culture was 14.8% with a range between farms of 9.4–45.5%. A higher prevalence rate of 35.7% was detected by quantitative PCR (qPCR) on a Slovenian middle-size dairy farm and in a Belgian veal calf farm (16% at the age of 18 days) [11,12]. In 603 German farms, a higher prevalence of 17.6% was detected, and similar to our study, used selective enrichment culture [9].

Based on recent data from a study in animal husbandry and agricultural bio-gas plants, where the prevalence of *C. difficile* reached 44.8% [33], it was expected that *C. difficile* prevalence could be higher on farms operating a bio-gas plant. In our study, the occurrence of *C. difficile* was not significantly more frequent in farms operating a bio-gas plant (*p* = 0.834) but the use of the digestate from this technology as bedding for cows was associated with the occurrence of *C. difficile* (*p* = 0.0026). The role of digestate in the *C. difficile*-life cycle in livestock is supported by a recent study that revealed the presence of *C. difficile* in raw digestate from five bio-gas plants in France [34]. 

Similar to our study, a higher risk of colonisation by *C. difficile* in calves of the Hostein-Friesian breed was found in the study by Bandelj et al. (OR 2.82, 95% CI 1.23–6.47; *p* = 0.014), [12]. Surprisingly, the study by Zidaric et al. that had a high *C. difficile* prevalence rate of 16%, sampled a cohort of Blue/Holstein-Friesian calves [11].

In our study, all but one of the *C. difficile* isolates belonged to the ST11 and RT033 predominated (84.1%). The predominance of RT033 in calves was also observed in Germany (57%) and Slovenia (75.5%) [9,12] and its presence in the gastrointestinal tract of calves is commonplace [31,32,35].

A MLVA, the method used for the determination of genetic relatedness also in other *Clostridia* [36], revealed clonal relatedness between *C. difficile* isolates of the same ribotype within and between individual farms. There were no records on the transport of animals between the farms in the study. Intra-farm relatedness of RT033 *C. difficile* isolates was also observed in the study of Bandelj et al. [37]. Clonal groups of ST 11 *C. diffcile* isolates from different states, countries and continents were also identified by core genome single nucleotide variant analysis suggesting possible zoonotic/anthroponic transmission [15].

The *C. difficile* ribotypes 046 and 126 belong to ribotypes identified in stool samples from CDI hospitalised patients in the Czech Republic and Europe as well [18,38]. The role of the *C. difficile* ribotype 033 in human CDI still remains unclear due to the absence of toxins A/B [8,16]. In humans, the RT033 CDI cases are rarely detected [39] and can be underdiagnosed using the recommended CDI laboratory diagnostics algorithm [40]. In our study, the phenotype expression of TcdA was not investigated and the detection of the *tcdA* gene fragment is more likely due to a different pathogenicity locus organisation as previously described in RT 033 by Rupnik et al. [16].

In this study none of the *C. difficile* isolates exhibited a resistance to metronidazole and vancomycin, CDI treatment drugs, but six *C. difficile* isolates (RTs 126 and 033) showed resistance to moxifloxacin, which is monitored through a standardized CDI surveillance protocol in humans [41]. Fluoroquinolone resistance, due to an amino-acid substitution Thr82Ile in the DNA gyrase, which weakens the interaction between quinolones and the enzyme [42], plays a role in human CDI epidemiology due to the spread of certain ribotypes [43]. The moxifloxacin resistance, caused by Thr82Ile, was also found in the ST11 *C. difficile* isolates derived from animals [15,17], and suggests that the monitoring of moxifloxacin-resistant *C. difficile* together with use of fluoroquinolones in agriculture is needed.

## 5. Conclusions

The Czech calves of the Holstein breed from farms using the digestate from bio-gas plants are more likely to be colonised by clonally-related *C. difficile* belonging to the sequence type 11 represented by ribotypes 033 and 126. The identified resistance to moxifloxacin with the amino-acid substitution Thr82Ile in the GyrA and other accessory antimicrobial resistance genes stress the need for further monitoring by the “One health approach”.

## Figures and Tables

**Table 1:** The characterisation of *C. difficile* isolates in the study. CLD: clindamycin; CIP: ciprofloxacin; ERY: erythromycin; MOX: moxifloxacin; TET: tetracycline; ST: sequence type.

Farm	No. of Samples Positive/Tested (%)	Ribotype	ST/Clade	Toxin Genes	Antimicrobial Resistance	Antimicrobial Resistance Determinants
**A**	3/32 (9.4)	033 (*n* = 2)	11/5	*tcdA*, *cdtA/B*	CIP (2); TET (1); CLD (2)	*tetA/B* (TET)
		046 (*n* = 1)	35/1	*tcdA*, *tcdB*	CIP (1); ERY (1); CLD (1); TET (1)	*ermB* (ERY and CLD)
**B**	5/19 (26.3)	126 (*n* = 5)	11/5	*tcdA*, *tcdB*, *cdtA*, *ctdB*	CIP (5); ERY (5); CLD (4), TET (2); MOX (5)	Thr82Ile (MOX)
**C**	2/11 (18.2)	033 (*n* = 2)	11/5	*tcdA*, *cdtA/B*	CIP (2); CLD (1)	-
**D**	3/16 (18.8)	033 (*n* = 3)	11/5	*tcdA*, *cdtA/B*	CIP (3); CLD (2); TET (1)	*tetA/B* (TET)
**E**	1/7 (14.3)	033 (*n* = 1)	11/5	*tcdA*, *cdtA/B*	CIP (1)	-
**F**	1/3 (33.3)	033 (*n* = 1)	11/5	*tcdA*, *cdtA/B*	CIP (1); CLD (1)	-
**G**	1/5 (20.0)	126 (*n* = 1)	11/5	*tcdA*, *tcdB*, *cdtA*, *ctdB*	CIP (1)	-
**H**	20/44 (45.5)	033 (*n* = 20)	11/5	*tcdA*, *cdtA/B*	CIP (17); ERY (1); CLD (6); MOX (1)	Thr82Ile (MOX)
**I**	7/29 (24.1)	033 (*n* = 7)	11/5	*tcdA*, *cdtA/B*	CIP (7); CLD (4); TET (2)	*tetA/B* (TET)
**J**	1/7 (14.3)	033 (*n* = 1)	11/5	*tcdA*, *cdtA/B*	-	-

**Table 2:** Risk factors associated with *Clostridioides* (*Clostridium*) *difficile* carriage in calves. CI: confidence interval.

Risk Factors	Odds Ratio	95% CI, Low	95% CI, Up	*p* Value
Bio-gas plant on the farm	5.3333	0.9282	30.6454	0.0834
Usage of digestate for bedding	27.0000	2.5034	291.1988	0.0026
Mastitis therapy in dry period	1.4286	0.2233	9.1376	1.0000
Type housing of calves	0.5926	0.0534	6.5719	0.2680
Type housing of cows	2.1250	0.2519	17.9273	0.5920
Breed of dairy cows (Holstein)	8.5000	1.2471	57.9331	0.0302
Size of cow herd > 200 heads	1.6970	0.3323	8.6661	0.6942

## Supplementary Materials

The following are available online at https://www.mdpi.com/2076-2607/8/6/901/s1, Table S1: Overview of farms included in the study; Table S2: Primers used in the study; Table S3: Overview of characterisation of *C. difficile* isolates in the study.
